# Late Pleistocene pottery production and exchange: Provenance studies of hunter-gatherer wares from southern Kyushu, Japan by neutron activation analysis

**DOI:** 10.1371/journal.pone.0265329

**Published:** 2022-03-16

**Authors:** Fumie Iizuka, Jeffrey R. Ferguson, Masami Izuho

**Affiliations:** 1 School of Social Sciences, Humanities and Arts, University of California, Merced, CA, United States of America; 2 Faculty of Social Sciences and Humanities, Tokyo Metropolitan University, Hachioji, Tokyo, Japan; 3 Department of Anthropology and Research Reactor Center, University of Missouri, Columbia, MO, United States of America; Universita degli Studi di Milano, ITALY

## Abstract

Late Pleistocene hunter-gatherers in East Asia adopted pottery, yet the ability to reconstruct circulation, mobility, and exchange has been hampered, in part, due to problematic regional geochronology. The driving forces behind pottery adoption is unclear. The purpose of this study is to test our results of the first systematic petrographic pottery sourcing from the pre-Younger Dryas by utilizing neutron activation analysis. We examine samples from the Sankauyama I site on Tanegashima Island, southern Japan, dating to the Incipient Jomon, ca. 14,000/13,500–12,800 cal BP, with a well-defined geochronology. Our NAA results corroborate with the petrographic study suggesting that pottery was mainly produced in-situ, but some vessels were transported long distances from another island. Changing from high mobility, sedentary Incipient Jomon foragers made pottery, occasionally investing in long-distance ceramic vessel transportation and exchange likely involving ocean crossing. This may be associated with a risk-buffering strategy in the context of rising sea levels and isolation of Tanegashima.

## Introduction

Production and circulation of the first pottery vessels adopted by hunter-gatherer groups of the Holocene have been well investigated in some regions, providing understanding of pottery’s relations to degrees of sedentism, mobility patterns, and exchange, and behavioral organizations [1–4]. For example, with the earliest pottery from the Savannah River basin of the Atlantic American Southeast (Early Stallings phase, ca. 5150–4100 cal BP) [5] foragers living in small communities transported vessels along rivers through seasonal subsistence mobility as well as exchange networks [4]. In the western Great Basin of North America, a clear adoption of ceramic technology is found from ca. 700–500 cal BP [1, 6] where highly mobile to less mobile small forager groups adapted to arid environments transported vessels to locations with distinct rainfall [2], and sometimes cached pottery in predictable resource zones [1]. In the Libyan Sahara, hunter-gatherer-fishers of the Late Acacus (ca. 10,200–8000 cal BP) adopted pottery during a humid period [7] with one study suggesting long-distance pottery circulation in the context of seasonally residentially mobile hunting and gathering [3]. Despite these scattered results from Holocene contexts, sourcing of late Pleistocene pottery produced and used by foragers have been rare and distribution mechanisms remain unclear. Pottery, a traditional signature of the Neolithic (e.g., [8]), is unsuitable for mobile foraging activities because it is fragile and heavy and requires at least some sedentary days to produce [1, 9]. Reasons behind the adoption of new technology need to be investigated.

According to radiocarbon-based geochronology, pottery vessel technology first appeared in East Asia and Northeast Asia in the late Pleistocene [10–16]. Degrees of mobility have often been suggested as ranging between high residential mobility to decreased mobility in the context of a broadening diet (e.g., [12, 17–19]). No systematic tests on mobility, however, have been done with ceramic assemblages from this period. Other than the Japanese archipelago and the Russian Far East, only small number of Pleistocene pottery-bearing sites have been discovered (e.g., [12, 20]) making the souring, and systematic reconstruction of production and circulation difficult. Furthermore, there are geochronological uncertainties in the key regions of East and Northeast Asia where pottery production began that inhibit further research. For example, in South China, AMS-^14^C dates have yielded the earliest geochronology in the world for pottery vessels, ca. 20,000–17,000 cal BP, [10, 19, 21–25] but earlier contextual data and their interpretations ([semi-] domesticated plant foods, thermoluminescence dates, and diagenesis) suggest dates can be as late as the Pleistocene-Holocene boundary [12, 20, 26–30]. Similarly, in the Transbaikal, dates centered on the AMS technique suggest the early pottery vessels are associated with ca.14,770/14,000–10,500 cal BP [14, 31–34]. However, a stratigraphic observation of depositional contexts and diagenesis give potential dates as young as 7,000–6,000 years ago [12, 35] or ca. 8,800–5,500 cal BP [31]. Reconstructing pottery production and distribution patterns in these regions should enhance our understanding of decisions made by hunter-gatherers who adopted and used ceramic vessels as part of an adaptation to terminal Pleistocene conditions. For the sourcing study, a careful selection of sites and regions with confident geochronology is required.

**Fig 1:**
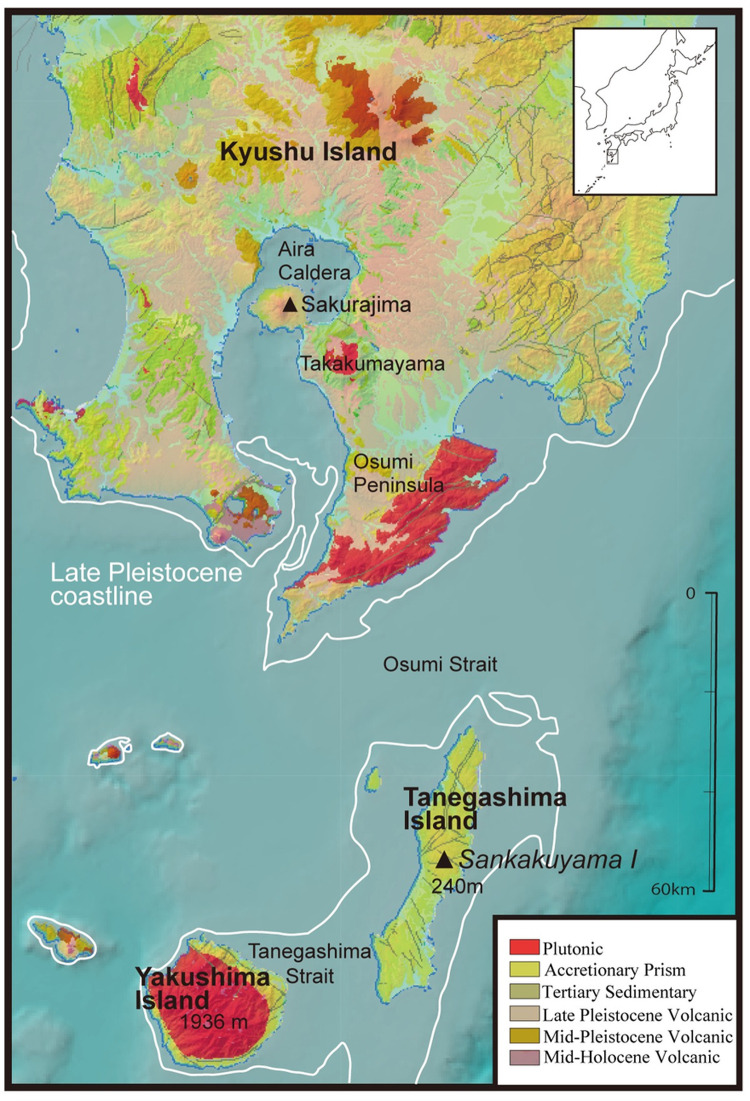
A geological map of southern Kyushu with main geographical place names and a site mentioned in the text. The lines in the ocean are inferred paleo-coastlines from around 14,000 cal BP. The map was reprinted/modified from GeomapNavi [43] and Iizuka and Izuho [38] (Fig 2), and Iizuka et al. [41] (Fig 1) with the original source, Environment Simulation Laboratory Co, Ltd. [44], under CC BY licenses, with permissions from the Geological Survey of Japan with the original copyright (2001–2019), Elsevier with the original copyright (2017 and 2022), and Environment Simulation Laboratory Co, Ltd. with the original copyright (2019–2021).

Southern Kyushu of southern Japan is an exceptional place from a geochronological perspective (Fig 1). This region has experienced recurring volcanic eruptions throughout the Quaternary, accumulating dated tephra layers [36]. Satsuma tephra (Sz-S) with tephrochonology of ca. 12,800 cal BP is found right above the first ceramic-bearing Incipient Jomon occupations, ca. 14,000/13,500–12,800 cal BP [37–39]. Many of the over 88 Incipient Jomon sites [40] are directly associated with Sz-S tephra. Those that do not have a clear tephra deposition have ceramic and lithic stylistic cross dates with sites having an intact Sz-S tephra layer. Sites in this context can be used to begin to reconstruct ceramic production and circulation patterns. Additionally, geological heterogeneity (Fig 1) makes this region [41] an appropriate location for sourcing. In this paper, we study pottery from the Sankakuyama I site of Tanegashima Island, off the southern coast of mainland Kyushu, and analyze sherds by neutron activation analysis (NAA). Our study tests the first systematic pottery sourcing done by petrographic analysis and electron microprobe on pottery prior to the Younger Dryas (starting ca. 12,900 cal BP, [42]) in East and Northeast Asia [41]. The NAA study from Sankakuyama I employs the same specimens used in the previous petrographic study.

With outstanding accuracy and precision, NAA is among the most powerful bulk geochemical techniques used in pottery provenance studies [45, 46]. NAA has been applied to ceramics in a variety of geographical regions, time periods, and socio-political and economic contexts (e.g., [2, 4, 47–52]). Furthermore, methods combining petrography and ceramic geochemistry have helped interpret production and circulation patterns [47, 48, 53, 54].

In this paper, we assess results on production zones, extent of production, circulation patterns, degrees of sedentism, and mobility and exchange. Sea level rise disconnecting Kyushu proper (hereafter, signifying the southern region of the Kyushu Island) and Tanegashima likely occurred by ca. 14,300 cal BP [38, 41, e.g., 55] during the Bølling/Allerød warm period, ca. 14,700–12,900 cal BP [42]. We place our interpretations on forager decisions in the context of the late Pleistocene environmental change.

### Archaeological context

Although the timing and nature of changes require further investigation, existing data suggest that varied behavioral changes occurred during terminal late Upper Paleolithic (LUP) and Incipient Jomon transitions in southern Kyushu [56]. Microblade technology was adopted by the Oldest Dryas, ca. 17,000/16,000 cal BP [57]. Although very high mobility is generally expected with microblade use [58], raw materials in Kyushu proper are suggested to have been procured from within 50 km [57]. Small assemblages of ceramics are found ambiguously associated with the microblade-using occupation by ca. 15,000 cal BP in Kyushu proper [12]. By the Incipient Jomon in southern Kyushu, the number of sites increased, and pit-dwellings, large grinding stones, probable updraft hearths, and greater amounts of pottery are found. Some Kyushu proper sites have microblades associated with this context [12, 37, 59]. The Incipient Jomon of Tanegashima, however, has more indicators of sedentism, with the addition of ground stone projectile points, substantially larger amounts of pottery, and a lack of microblades (e.g., [12]). There are 11 Incipient Jomon sites identified so far from Tanegashima [60], doubling in number from the terminal LUP sites with signatures of high mobility including microblade use and features without investments (e.g., [61]).

**Fig 2:**
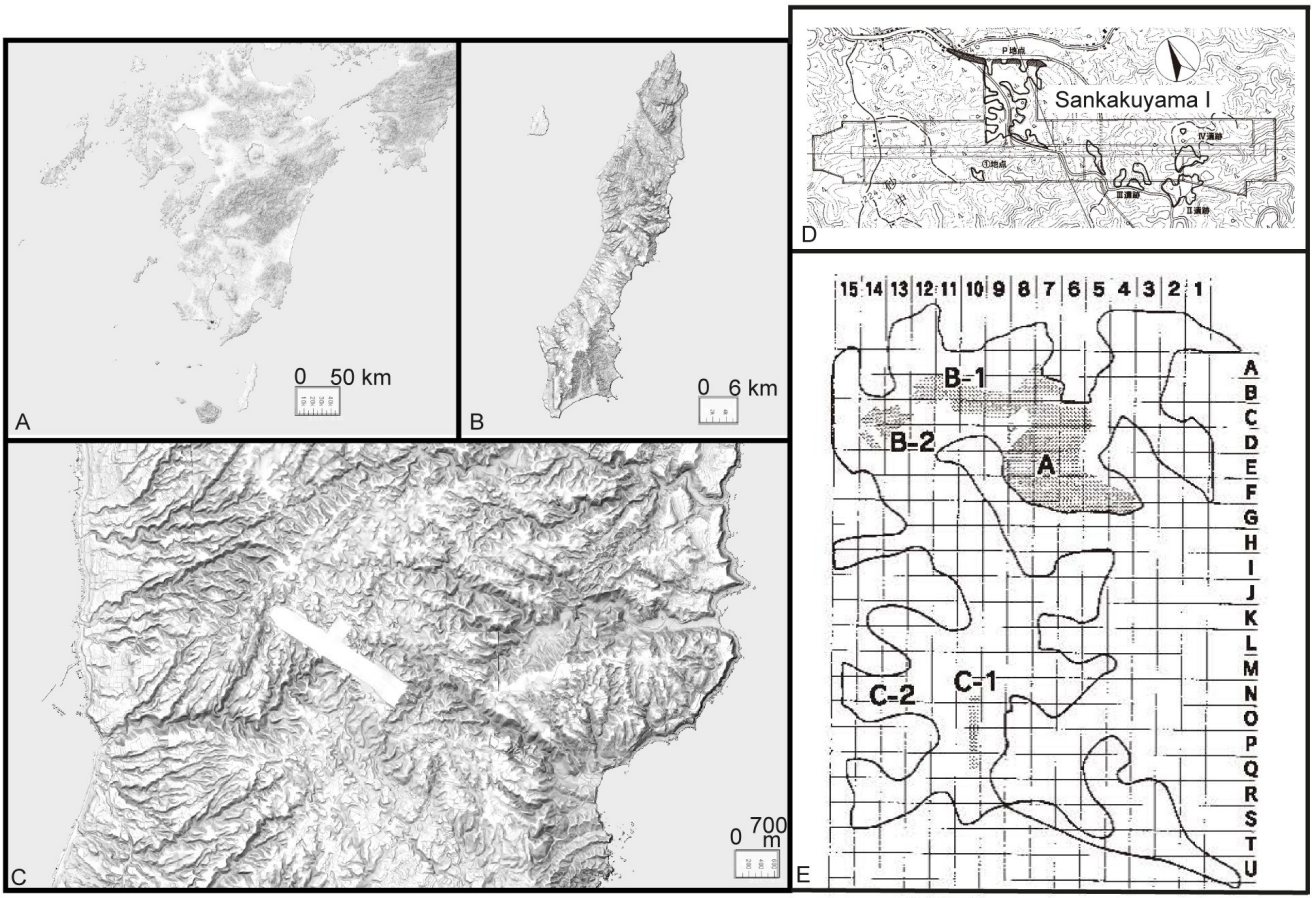
Maps of Tanegashima Island and Sankakuyama I. The map was reprinted/modified from Iizuka et al. [63], Tomohiko Sugimoto [64] of Kashmir 3D, and Geospatial Information Authority of Japan [65] under CC BY licenses, with a permission from the Kagoshima Prefectural Archaeology Center, original copyright (2006), Tomohiko Sugimoto of Kashmir 3D, original copyright (1994–2006), and the Geospatial Information Authority of Japan, original copyright (2016). A: A map that shows the position of Kyushu Island, Tanegashima Island, and Yakushima Island. B: Tanegashima Island. C: A map showing the location of Sankakuyama sites on Tanegashima. D: The site boundary of Sankakuyama I. E: The excavation grid of Sankakuyama I.

The Sankakuyama I site of the Tanegashima Island was excavated extensively (an area of 58,620 m^2^) by the Kagoshima Prefectural Archaeology Center [62] (Fig 2). The site is located on a high marine terrace. It is an open-air, multi-component site with Incipient, Initial, and Early Jomon, and Kofun occupations [12, 62]. The Incipient Jomon occupation is found in layer V, right below primary depositional unit of Sz-S tephra. Radiocarbon dates range between ca. 14,000–13,570 cal BP and 11,030–9520 cal BP; however, with the depositional position below the well-dated Sz-S tephra, date ranges are suggested to be 14,000/13,500–12,800 cal BP [41].

**Fig 3:**
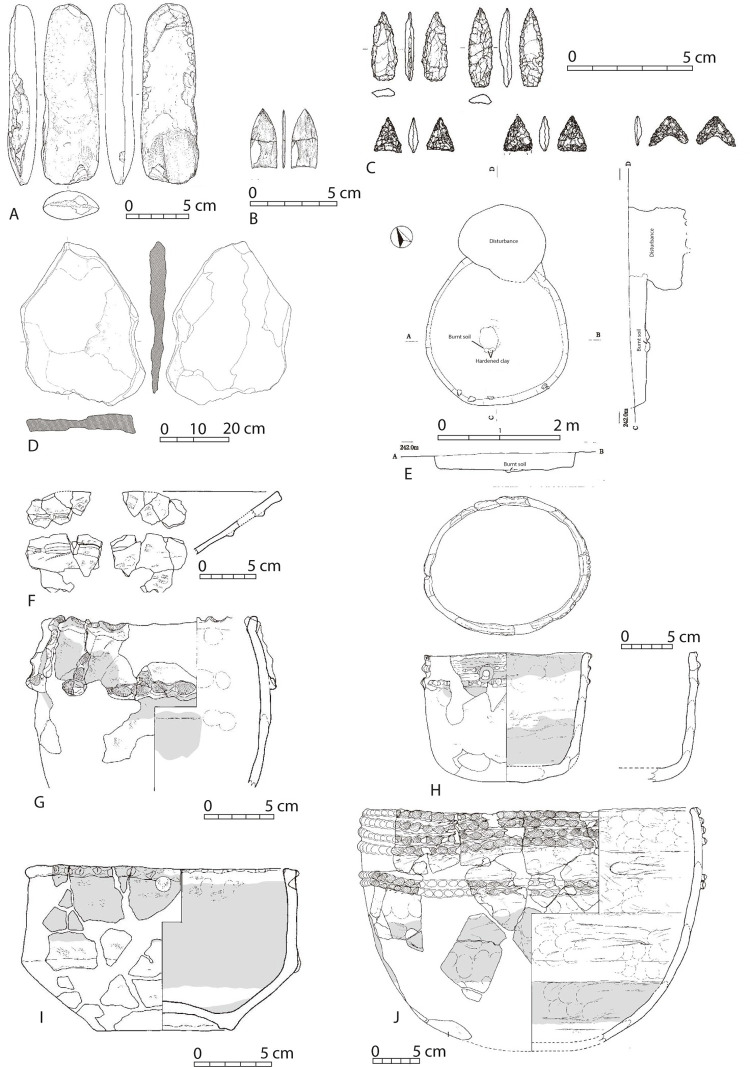
Artifacts and features associated with the Incipient Jomon period at Sankakuyama I. The images in this figure are reprinted and modified from figures in Kagoshima Prefectural Archaeology Center [62] under a CC BY license, with a permission form the Kagoshima Prefectural Archaeology Center, original copyright (2016). A: ground stone axes. B: ground projectile point. C: projectile points (above), small projectile points or arrowheads (below) D: ground stone, E: A pithouse with burnt soil and hardened clay in the center. F: to J are drawings of reconstructed ceramic vessels.

The Incipient Jomon pottery assemblage includes approximately 4000 sherds. Vessel forms are deep to shallow somewhat closed to open mouthed bowls. Decorations are mostly applique bands. Lithics include flaked arrowheads, ground arrowheads, wedge-shaped tools, secondary retouched flakes, cores, ground stone axes, stone plates, grinding stones, pebble tools, hammerstones, and polished stones [12, 41, 62, 63]. Features include pit-dwellings, pit aggregates, earth pits, pebble aggregates, a flaked stone production area, and areas with burnt soil (Fig 3).

### Geographical, paleoenvironmental, and geological contexts

Southern Kyushu comprises the current day Kagoshima and Miyazaki Prefectures of the southern regions of Kyushu Island and a chain of numerous islands extending south with Yoronjima Island as the southern boundary. Kyushu proper, Tanegashima, and Yakushima are the islands most significant to this provenance study and are individually described below. More detailed geographical, paleoenvironmental, and geological contexts of this region are provided in Iizuka et al. [41].

In Kyushu proper, the northern part of Kagoshima Bay includes the Aira Caldera with the active Sakurajima Volcano. A minimum distance between Tanegashima and the Osumi Peninsula is about 33 km and between Tanegashima and Yakushima is about 17 km. Tanegashima has low hills with a maximum elevation of 282 m.a.s.l. In contrast, the mountainous Yakushima has a maximum elevation of about 2000 m.a.s.l. and has more diverse vertical ecological zones. Most of Kagoshima proper and the northeastern Tanegashima have an annual average precipitation range of about 2000–2800 mm, but the southern tip of the Osumi Peninsula, southern Tanegashima, and the lowland Yakushima have a higher range of about 3000–4000 mm [41].

The southern tip of Kyushu Island, Tanegashima, and Yakushima, are inferred to have been warmer than the northern part of southern Kyushu. Mainly due to the warm ocean current, southern Kyushu had a warm climate even during the Last Glacial Maximum, ca. 26,500–19,000 cal BP (e.g., [66, 67]) with warm-temperate species and temperate coniferous forest dominated by temperate deciduous broad-leaved mixed forest [38, 41]. The lowlands of the southern tip of Kyushu proper, then connected with Tanegashima and Yakushima, additionally had warm-temperate evergreen forests/broad-leaved evergreen forests as refugia [38, 41, 68, 69]. Yakushima, with a higher elevation, had additional biomes not present on Tanegashima. The onset of the Incipient Jomon occupation roughly corresponds with the sea level rise inferred from research in the northern Kagoshima Bay (e.g., [55]). Tanegashima may have been disconnected from Kyushu Island by 14,300 cal BP, with the emergence of the Osumi Strait, and during the Incipient Jomon, Tanegashima Strait may have appeared [41].

In Kyushu proper, mudstone and sandstone units of the Late Cretaceous lower (northern) Shimanto belt are found in the southwest and central Satsuma Peninsula, areas surrounding Takakumayama, and in northwestern Kagoshima Bay [70, 71]. Northeastern, central, and the southwestern Osumi Peninsula have mudstone and sandstone units of the upper (southern) Shimanto supergroup from the Paleogene [70, 72, 73]. There is a wide distribution of volcanic and tephra sediments above these sedimentary belts [41, 70, 71]. In the Osumi Peninsula, a granitic unit from the mid-Miocene intrudes in Takakumayama, and the large area in the southern Osumi Peninsula [41]. There are additional small granitic units in the Satsuma Peninsula. Tanegashima and Yakushima geology relate to the upper Shimanto supergroup classified as Kumage group [74]. On Yakushima, there is a major intrusive granitic unit (Fig 1). Moreover, there is middle to late Pleistocene distal tephra from the Quaternary, Kakutou (Kkt), Ata (Ata), Kikai-Tozurahara (K-Tz), and Aira Tn (AT) tephra found in Kagoshima proper. On Tanegashima, Ata, K-Tz, Tane I-IV, and AT tephra and other late Pleistocene tephra including Sz-S are reported. The Koseda tephra of ca. 0.58 Ma is additionally reported from Yakushima [74]. Tanegashima Island has no plutonic, obsidian, or andesite outcrops and their absence facilitate distinction of locally produced and exotic artifacts recovered from the island context.

### Previous research results

**Fig 4:**
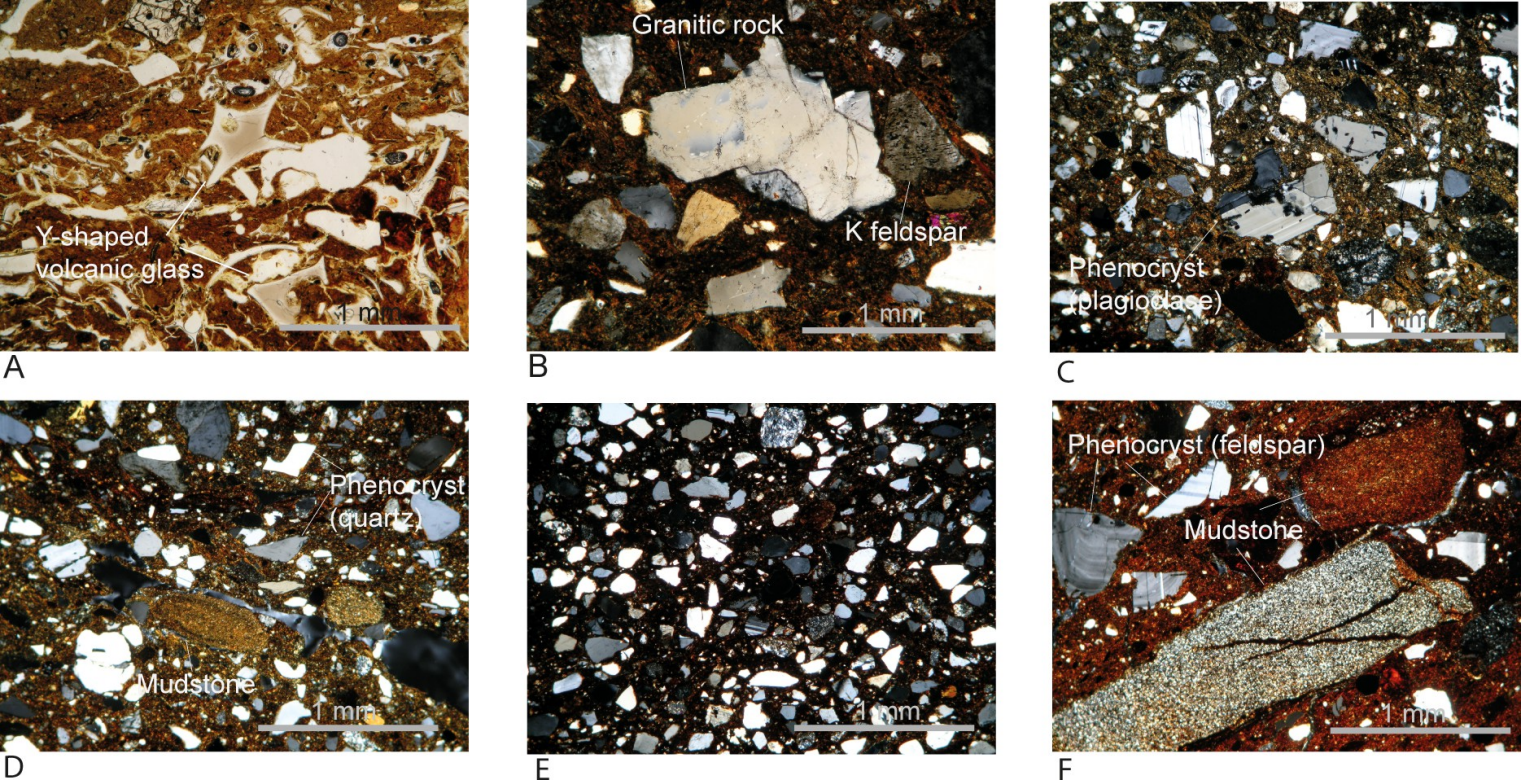
Examples of sourced petrographic groups. Images are reproduced/modified from Fig 4 of Iizuka et al. [41] under CC BY licenses, with permissions from Elsevier with the original copyright (2022). We also added new images previously unpublished. Petrographic thin sections were examined by polarized microscopes and tests were done through an analysis by an electron microprobe [41]. A: Type 1 exemplified by SNK-I-001 (PPL) rich in volcanic glass from AT tephra. B: Type 2 exemplified by SNK-I-041a (XPL) with angular granitic fragments and associated minerals. C and D: Type 3 exemplified by SNK-I-054 and SNK-I-002 (XPL) dominated by single grains of phenocrysts with lesser amounts of sedimentary rocks. E: Type 4 exemplified by SNK-I-014 (XPL) with sandstone-like composition. F: Type 5 exemplified by SNK-I-027 (XPL) with sedimentary rocks and single grains of phenocrysts.

Visual analyses of the formal variability of pottery have been previously published [38, 41, 63]. Here we describe the results from pottery (n = 58) and raw material (n = 50) thin section analysis using polarized microscope (Fig 4) and electron microprobe. Thin sections studied by microprobe (n = 3) are from three most distinct sources identified by the polarized microscope studies [41]. Pottery sources were classified into five types. Type 1 (n = 2, Fig 4A) is dominated by Y-shaped volcanic glass, identified as derived from AT tephra. Although there are AT tephra layers on Tanegashima, they are thin, around 20 cm. Instead, about 30 km southeast of Sakurajima volcano, AT tephra is densely deposited, at least to 800 cm. We suggest that these sherds may have been from the northern part of the Osumi Peninsula. Type 2 (n = 6, Fig 4B) is rich in bedrock-derived granitic rocks and associated minerals, likely coming from Yakushima or the southern Osumi Peninsula. These are clearly imported vessels. Type 3 (n = 36, Fig 4C and 4D) is composed of major amounts of single grains of phenocrysts derived from tephra, lesser amounts of sedimentary, and trace amounts of plutonic rock fragments. Type 4 (n = 12, Fig 4E) has rocks and mineral inclusions similar to the composition of sandstones available locally. Type 5 (n = 2, Fig 4F) has abundant single grains of phenocrysts and sedimentary rock fragments [41]. Because the majority of sherds have locally produced signatures, and the production patterns match the high degrees of sedentism indicated at Sankakuyama I with pit-dwellings, heavy duty grinding stones, and other ground stone implements, it is suggested that residents had high degrees of sedentism, producing pottery in situ. The proportion of exotic pottery, from outside the present-day Tanegashima Island, is small, between 10–14%, observed in the thin sectioned samples. This is unlike the small flake-based tools of foreign origin, of obsidians and andesites, and probably also chert, estimated to be between 30–40% [41, 75].

### NAA and behavioral expectations

We hypothesize that paralleling the results from petrography, samples will be discriminated into the same source groups with NAA. The results should show clear differences among ceramic Types 1 and 2, that are petrographically quite distinct, from Types 3, 4, and 5 that are likely locally produced ware. Among Types 3, 4, and 5, there will be compositional overlaps, but there will be geochemical differences.

Geological literature on geochemical compositions of bedrocks and variability by geological units is scarce in southern Kyushu. However, from inclusion types observed in thin sections, we expect that the NAA-based geochemistry of pottery should detect elevated K (potassium) concentrations in petrographic Type 2 samples rich in near bedrock-derived granitic rocks and K-feldspars. With feldspar rich characteristics, these samples are also expected to have higher Al than volcanic glass-rich Type 1. Similarly, we suggest that Type 2 should have more elevated Al than sherds of Types 3 and 5 with mudstones, pyroclastics, and sandstones contained in distinct proportions as inclusions, and Type 4 with sandstones.

Increased sedentism is expected in the context of resource predictability and concentration among foragers [76], but some level of exchange is expected even in those conditions [41, 77, 78]. At Sankakuyama I on Tanegaghima Island with relatively warm and predictable environment in proximity to the coast (e.g., [79]) during the Incipient Jomon, this expectation of sedentism is reasonable. Although exotic small flake-based tools found in much higher proportion at Sankakuyama I requires alternative explanations, the evidence of only some exotic pottery (up to 14%) obtained from the petrographic results align with this perspective. Hunter-gatherers engage in feasting, communal hunting, trading, and information exchange through inter-band, supra-band, and regional networks [77, 80] in part to buffer risks of resource failures and loss of crucial information for sustenance (e.g., [18, 81–85]). Because pottery production during logistical foraging in remote places and transporting vessels back to Sankakuyama I where they already have vessels is costly, we infer that the imported ware from other islands found at Sankakuyama I relates to social networks and exchange with other groups [41].

## Materials and methods

A necessary permit was obtained for the described study, which complied with all relevant regulations. We received permission from the Kagoshima Prefectural Archaeology Center for a minimally destructive analysis. The archaeological center previously analyzed ceramic sherds (total of about 4000) excavated from Sankakuyama I, adopting their classification criteria combining thickness, production techniques, decorative styles, and paste types. After considering horizontal and vertical provenience and proximity within the excavation contexts, they reconstructed vessels using those sherd fragments. There were nine complete vessels. Six additional vessels had enough fragments for the vessel forms to be reconstructed [62]. After the reconstruction, there were 3303 sherds and vessels, which were classified into 15 types. Samples that did not go through reconstructions were placed by the archaeology center into distinct bags based on their classification criteria. We studied sherds that were not parts of reconstructions. We examined sherds from all bags and selected sherds (n = 58) from different bags and from sherds that exhibited the most diverse paste and technological variability based on visual observations [41, 63] as well as samples derived from diverse horizontal excavation contexts. In order to test the analytical results from the thin section analyses, the same sherd samples were chosen for the NAA analysis. A minimum of 2 grams were removed from each sample. These samples were sent to the Archaeometry Laboratory at the University of Missouri. Sample preparation, irradiation, and data collection were carried out using standard procedures presented in detail elsewhere [46]. Using the pneumatic tube irradiation system (flux of 8 x 10^13^ neutrons cm^-2^ s^-1^) and sample standards in polyethylene vials, we irradiated and measured short-lived elements (Al, Ba, Ca, Dy, K, Mn, Na, Ti, and V) for 5 seconds and decayed for 25 minutes; we counted for 12 minutes. Medium-lived elements (As, La, Lu, Nd, Sm, U, and Yb) and long-lived elements (Ce, Co, Cr, Cs, Eu, Hf, Ni, Rb, Sb, Se, Sr, Ta, Tb, Th, Zn, and Zr) were irradiated in the reactor pool (flux of 5 x 10^13^ neutron cm^-2^ s^-1^) for 24 hours with standards. The medium-lived elements were decayed for 7 days and counted for 2000 seconds. Long-lived elements were decayed for 3 to 4 weeks and counted for 10,000 seconds [46]. We conducted one-way analyses using JMP and bivariate analysis with the MURR statistical routines in Gauss 8.0 [86]. We explored varied potential geochemical groups in the bivariate analysis by paying particular attention to the chemical groups that align with distinct groups visible through petrography.

## Results

The one-way and bivariate analyses discriminate samples into the following geochemical Groups 1 through 5.

**Fig 5:**
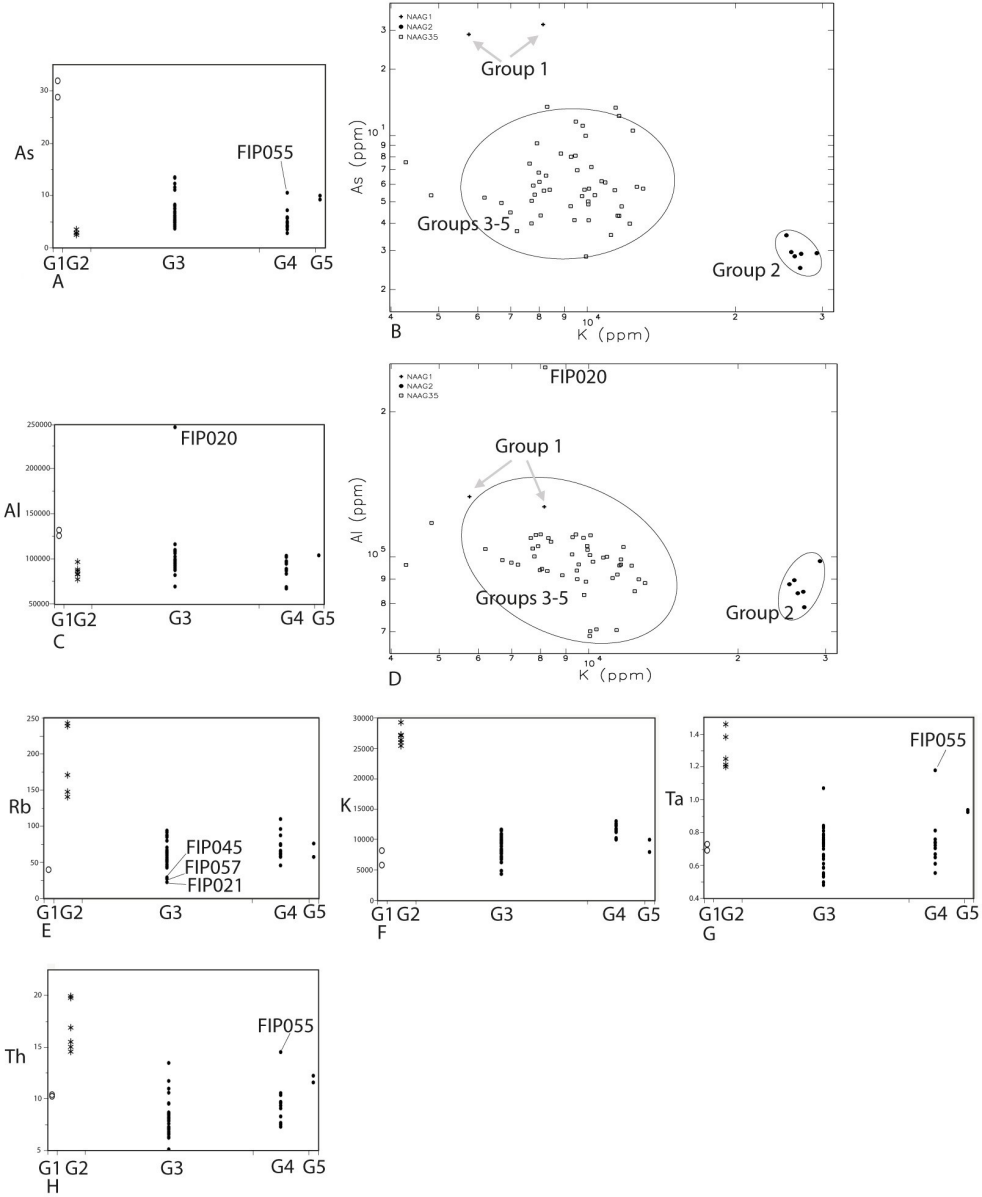
One-way analyses (A, C, and E to H) and bivariate plots (B and D) of distinct elements and discriminations of groups discussed in the text. The letter “G” in the x-axis of the one-way analyses stands for groups. The horizontal distance in the x-axis in the one-way analysis are the sample numbers in groups. As and Al are elevated in Group 1 shown in “A”, “B”, “C”, and “D”. K is elevated in Group 2 shown in “B”, “D”, and “F”. Rb, Ta, and Th are also elevated in Group 2, shown in “E”, “G”, and “H”. For the bivariate analyses with Gauss 8.0, ellipses are based on the confidence intervals at 90%.

In Group 1 (n = 2, Table 1), AT tephra-based sherds identified as Type 1 from petrographic analysis (n = 2) are clearly distinguishable from other groups and samples assigned to them, with a higher concentration of As in the one-way analysis (Fig 5A), and with As plotted against K in the bivariate analysis (Fig 5B). In the one-way analysis of Fig 5C excluding a single sample (FIP020) in Group 3, and in the bivariate plot of Fig 5D excluding a single sample (FIP020) in Groups 3–5, with an anomalously high level of Al (aluminum), Group 1 has somewhat higher level of Al than all other studied groups and samples. Also, Group 1 has lower level of Rb than most samples except for FIP045, 057, and 021 in Group 3 as shown in the one-way analysis (Fig 5E).

**Table 1:** Sourced Groups 1 to 5 by NAA compared with petrographic Types 1 to 5.

ID: NAA	ID: thin section	Vessel Form	Exterior Decoration	Provenience*	Type: petrography	Group: NAA
FIP001	SNK-I-001	Jar	None	Area A, Grid F-5, Layer V	1	1
FIP002	SNK-I-002	N/A	None	Area A, Grid F-5, Layer V	3	3
FIP003	SNK-I-003	Bowl	Applique	Ara A, Grid G-5, Layer V	3	3
FIP004	SNK-I-004	N/A	None	Area A, Grid F-5, Layer V	3	3
FIP005	SNK-I-005	N/A	None	Area A, Grid F-5, Layer V	3	3
FIP006	SNK-I-006	N/A	None	Area B-1, Grid F-5, Layer V	2	2
FIP007	SNK-I-007	N/A	None	Area A, Grid F-5, Layer V	3	3
FIP008	SNK-I-008	N/A	None	Area B-1, Grid B-12, Layer V	2	2
FIP009	SNK-I-009	Bowl	None	Area B-2, Grid C-12, Layer V	2	2
FIP010	SNK-I-010	N/A	None	Area B-1, Grid B-11, Layer V	2	2
FIP011	SNK-I-011	Bowl	Applique	Area A, Grid D-7, 2T, Layer V	4	4
FIP012	SNK-I-012	N/A	None	Area A, Grid B-8, Layer V	3	3
FIP013	SNK-I-013	Bowl	None	Area A, Grid F-6, Layer V	4	4
FIP014	SNK-I-014	N/A	None	Area A, Grid F-7, Layer V	4	4
FIP015	SNK-I-015	Bowl	Applique	Area A, Grid F-6, Layer V	3	3
FIP016	SNK-I-016	N/A	None	Area A, Grid F-7, Layer V	3	3
FIP017	SNK-I-017	N/A	None	Area A, Grid B-8, Layer V	3	3
FIP018	SNK-I-018	Bowl	Applique	Area B-1, Grid B-10, Layer V	3	3
FIP019	SNK-I-019	N/A	None	Area A, Grid C-8, Layer V	3	3
FIP020	SNK-I-020	N/A	None	Area A, Grid B-8, Layer V	3	3
FIP021	SNK-I-021	Bowl	None	Area A, GridB-8, Layer V	3	3
FIP022	SNK-I-022	N/A	None	Area A, Earth Pit 1, Layer V	3	3
FIP023	SNK-I-023	N/A	None	Area A, Grid B-8, Layer V	3	3
FIP024	SNK-I-024	Bowl	None	Area A, Grid D-6, Layer V	3	3
FIP025	SNK-I-025	Bowl	None	Area B-2, Grid C-13, Layer V	5	5
FIP026	SNK-I-026	Jar	None	Area B-2, Grid C-13, Layer V	4	4
FIP027	SNK-I-027	N/A	None	Area B-2, Grid D-13, Layer V	5	5
FIP028	SNK-I-028	N/A	Applique	Area B-8, Earth pit 1, Layer V	3	3
FIP029	SNK-I-029	N/A	None	Area A, Grid B-7, Layer V	4	4
FIP030	SNK-I-030	N/A	Applique	Area A, Grid D-6, Layer V	3	3
FIP031	SNK-I-031	N/A	None	Area A, Grid D-6, Layer V	3	3
FIP032	SNK-I-032	Bowl	Applique	Area A, Grid D-6, Layer V	3	3
FIP033	SNK-I-033	N/A	None	Area A, Grid D-6, Layer V	3	3
FIP034	SNK-I-034	N/A	None	Area A, Grid B-8, Layer V	4	4
FIP035	SNK-I-035	N/A	None	Area A, Grid B-8, Layer V	4	4
FIP036	SNK-I-036	Bowl	None	Area A, Grid B-8, Layer V	4	4
FIP037	SNK-I-037	Bowl	None	Area A, Grid D-6, Layer V	3	3
FIP038	SNK-I-038	N/A	None	Area A, Grid B-8, Layer V	3	3
FIP039	SNK-I-039	N/A	None	Area B-1, Grid C-10, Layer V	4	4
FIP040	SNK-I-040	N/A	None	Area A, Grid 4T, Layer V	1	1
FIP041	SNK-I-041a	N/A	None	Area B-1, Grid B-12-all, Layer V	2	2
FIP042	SNK-I-041b	N/A	None	Area B-1, Grid B-12-all, Layer V	2	2
FIP043	SNK-I-042a	N/A	None	Area A, Grid F-1T-all, Layer V	3	3
FIP044	SNK-I-042b	N/A	None	Area A, Grid F-1T-all, Layer V	3	3
FIP045	SNK-I-043	N/A	None	Area B-2, Grid D-14, Layer V	3	3
FIP046	SNK-I-044	N/A	None	Area B-1, Grid C-10, Layer V	4	4
FIP047	SNK-I-045	N/A	None	Area B-1, Grid C-10, Layer V	4	4
FIP048	SNK-I-046	Bowl	Applique	Area B-1, Grid B-10, Layer V	3	3
FIP049	SNK-I-047	N/A	None	Area B-1, Grid B-10, Layer V	3	3
FIP050	SNK-I-048	N/A	None	Area B-1, B-10, Layer V	3	3
FIP051	SNK-I-049	N/A	None	Area A, Grid D-6, Layer V	3	3
FIP052	SNK-I-050	N/A	None	Area A, Grid F-5, G-5, Layer V	3	3
FIP053	SNK-I-051	N/A	None	Area B-1, Grid C-10, Layer V	3	3
FIP054	SNK-I-052	N/A	None	Area B-2, Grid D-14, Layer V	3	3
FIP055	SNK-I-053	Bowl	None	Area B-1, Grid C-10, Layer V	4	4
FIP056	SNK-I-054	N/A	None	Area A, Grid D-6, Layer V	3	3
FIP057	SNK-I-055	Bowl	None	Area A, Grid B-8, Layer V	3	3
FIP058	SNK-I-056	N/A	None	Area A, Grid F-7, Layer V	3	3

These samples were excavated from the Sankakuyama I site (N30°36’, E130°59’) of Tanegashima Island, and are stored at the Kagoshima Prefectural Archaeology Center in Kagoshima Prefecture, Japan. Sherd ID in NAA, in thin sections, vessel forms, the type of decoration, and the sherd provenience are also provided. *The information is taken from Iizuka et al. [63].

Potassium clearly discriminates the samples in Group 2 (n = 6, Table 2) from all others in both bivariate and one-way analyses as shown in Fig 5B, 5D, and 5F. Samples in Group 2, have significantly elevated K compared to the rest of the samples. As found in S1 Table and Table 2, within Group 2, K has a similar range, 25369 to 29235 ppm (average 26860 ppm with a standard deviation 1346), with a low coefficient of variation of 5.01. The rest of the samples mostly group together within or are very close to the 90% confidence interval for the ellipse of Groups 3, 4 and 5 in both bivariate plots of K and As, and K and Al (Fig 5B and 5D). Also, in other one-way plots (Fig 5E, 5G and 5H), Rb, Ta (except for FIP055), and Th (except for FIP055) are higher in Group 2 than in samples of other groups.

**Table 2:** Quantitative geochemical values of elements analyzed with NAA and classified into groups.

Group	Group 1 (n = 2)			Group 2 (n = 6)			Group 3 (n = 36)			Group 4 (n = 12)			Group 5 (n = 2)		
Element	Mean (ppm)	Std. Dev. (ppm)	% Coefficient of Variation	Mean (ppm)	Std. Dev. (ppm)	% Coefficient of Variation	Mean (ppm)	Std. Dev. (ppm)	% Coefficient of Variation	Mean (ppm)	Std. Dev. (ppm)	% Coefficient of Variation	Mean (ppm)	Std. Dev. (ppm)	% Coefficient of Variation
As	30.298	2.198	7.255	2.938	0.330	11.239	6.657	2.611	39.227	5.291	2.027	38.320	9.592	0.526	5.485
La	29.681	0.491	1.653	38.880	22.533	57.955	31.397	18.832	59.980	26.371	13.617	51.637	21.529	5.109	23.732
Lu	0.400	0.028	7.030	0.409	0.133	32.620	0.393	0.171	43.464	0.380	0.155	40.703	0.435	0.012	2.842
Nd	25.033	0.784	3.131	31.826	15.992	50.247	27.829	19.005	68.291	24.203	11.826	48.861	21.425	1.321	6.165
Sm	5.595	0.280	5.012	6.480	2.988	46.102	5.659	3.431	60.623	5.005	2.407	48.082	5.046	0.492	9.756
U	2.591	0.352	13.577	3.834	0.592	15.441	2.562	0.960	37.455	2.731	1.085	39.733	2.795	0.037	1.323
Yb	2.610	0.087	3.333	2.698	1.046	38.776	2.626	1.244	47.387	2.485	1.102	44.351	2.779	0.093	3.364
Ce	28.494	0.895	3.142	66.509	26.015	39.115	31.612	7.927	25.076	37.677	16.016	42.509	47.480	7.419	15.626
Co	8.186	0.558	6.818	6.335	0.764	12.053	9.186	5.635	61.340	8.506	4.816	56.624	13.532	0.620	4.585
Cr	37.557	0.247	0.657	39.325	4.217	10.723	51.515	20.256	39.321	45.613	16.670	36.546	62.119	9.046	14.562
Cs	8.918	0.675	7.573	13.367	5.030	37.629	8.068	4.402	54.566	9.502	3.380	35.573	8.285	3.008	36.306
Eu	1.542	0.032	2.083	0.988	0.494	50.012	1.411	0.783	55.484	1.114	0.421	37.812	1.225	0.049	4.028
Fe	37264.967	140.203	0.376	26114.835	4570.388	17.501	36703.114	11511.858	31.365	28587.594	10756.413	37.626	57973.719	2239.871	3.864
Hf	6.198	0.066	1.058	8.245	0.350	4.243	5.321	1.773	33.326	8.156	6.409	78.575	7.152	0.149	2.086
Ni	0.000	0.000	0.000	0.000	0.000	0.000	2.420	10.430	430.893	1.652	5.723	346.410	0.000	0.000	0.000
Rb	38.761	0.361	0.930	178.898	48.096	26.885	57.664	16.881	29.275	70.227	18.221	25.945	65.438	13.135	20.072
Sb	0.673	0.080	11.942	0.405	0.049	12.010	0.548	0.133	24.211	0.496	0.100	20.171	0.643	0.120	18.573
Sc	23.472	0.011	0.046	13.658	1.074	7.861	19.996	3.040	15.203	16.453	4.162	25.295	22.058	1.075	4.872
Sr	146.919	50.581	34.428	33.068	39.411	119.182	103.134	38.372	37.206	65.914	35.275	53.517	55.953	79.129	141.421
Ta	0.709	0.027	3.741	1.291	0.104	8.061	0.690	0.124	17.995	0.732	0.157	21.379	0.929	0.009	0.974
Tb	0.799	0.138	17.267	0.711	0.399	56.159	0.847	0.573	67.594	0.628	0.352	56.152	0.566	0.132	23.387
Th	10.319	0.117	1.131	16.952	2.381	14.048	8.113	1.642	20.236	9.464	1.934	20.439	11.906	0.458	3.850
Zn	72.674	7.591	10.445	74.046	8.453	11.415	76.785	18.540	24.145	69.978	20.731	29.625	91.643	9.171	10.007
Zr	138.544	18.239	13.164	213.199	25.031	11.741	142.812	53.785	37.662	212.220	175.797	82.837	161.528	24.611	15.237
Al	130183.797	4440.631	3.411	87139.378	6494.275	7.453	103867.334	26252.582	25.275	88636.839	12842.438	14.489	105358.672	52.911	0.050
Ba	177.878	12.443	6.995	439.919	26.533	6.031	264.144	60.425	22.876	346.697	73.599	21.229	241.675	59.430	24.591
Ca	14031.200	1771.218	12.623	3305.997	988.230	29.892	10913.440	3834.732	35.138	5414.196	1699.618	31.392	10026.737	177.112	1.766
Dy	4.548	0.017	0.383	4.230	2.034	48.090	4.649	2.955	63.552	4.001	1.919	47.957	4.257	0.035	0.811
K	6946.405	1688.246	24.304	26859.796	1345.722	5.010	8707.326	1714.521	19.691	11366.040	1083.227	9.530	8922.334	1431.844	16.048
Mn	541.985	106.485	19.647	410.138	50.217	12.244	482.389	279.534	57.948	335.170	267.077	79.684	594.991	2.782	0.468
Na	11796.309	243.062	2.060	6453.546	2412.917	37.389	10794.661	2133.726	19.766	9172.134	1522.791	16.602	9801.461	239.510	2.444
Ti	4077.019	303.785	7.451	5025.720	302.255	6.014	5540.663	1463.556	26.415	4110.615	1411.068	34.327	6566.572	162.552	2.475
V	124.708	10.709	8.588	67.780	8.764	12.931	147.432	47.046	31.911	110.268	39.442	35.769	228.067	14.774	6.478

Mean (ppm), standard deviation (ppm) and % coefficient of variation are provided for Groups 1 to 5. The format of this table borrows the style from Table 3 in page 676 of [49].

**Fig 6:**
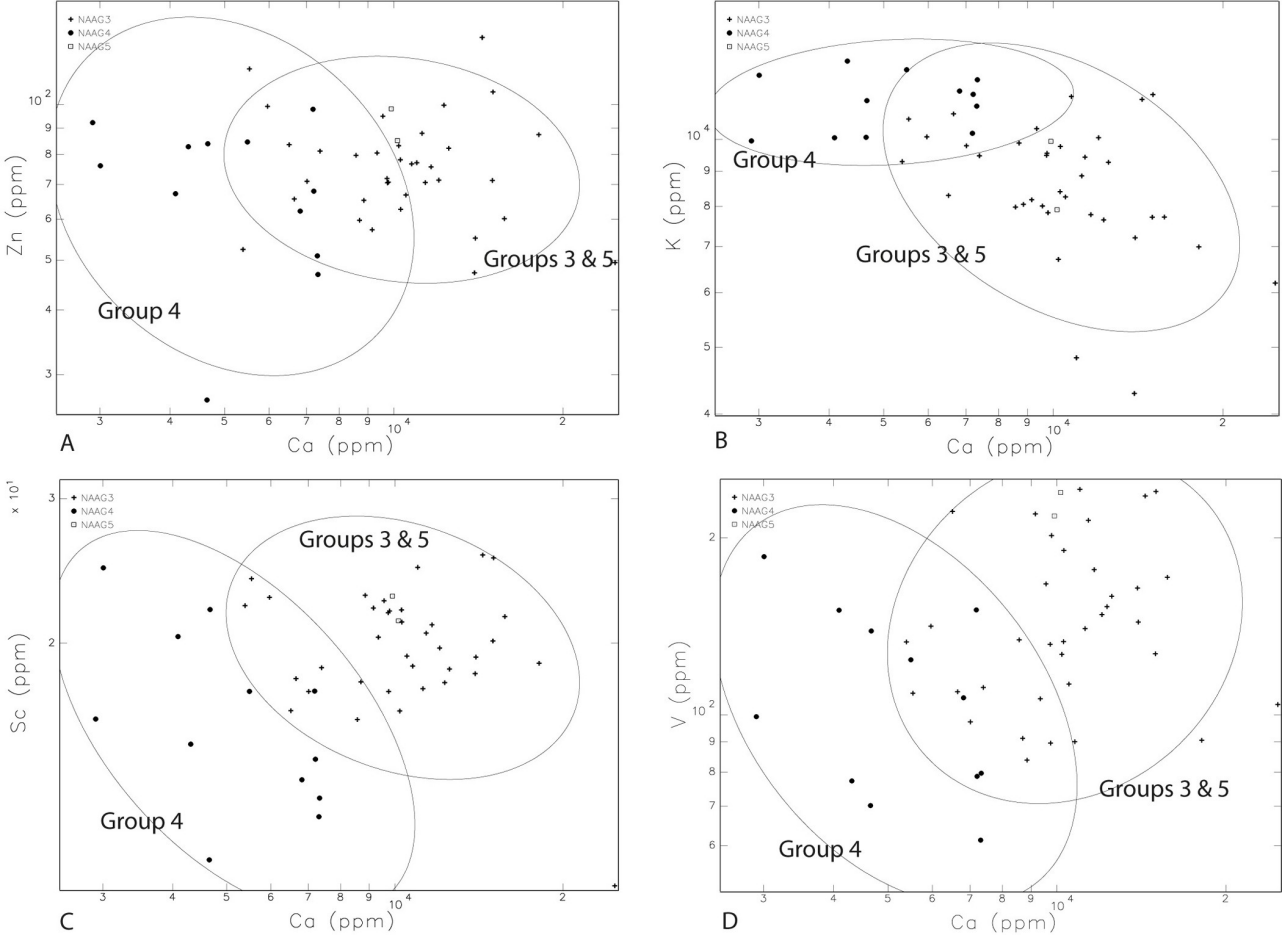
Bivariate plots A to D show that Group 4 and Groups 3 and 5 are discriminated with partial overlaps. “A” has Ca and Zn, “B” has Ca and K, “C” has Ca and Sc, and “D” has Ca and V.

**Fig 7:**
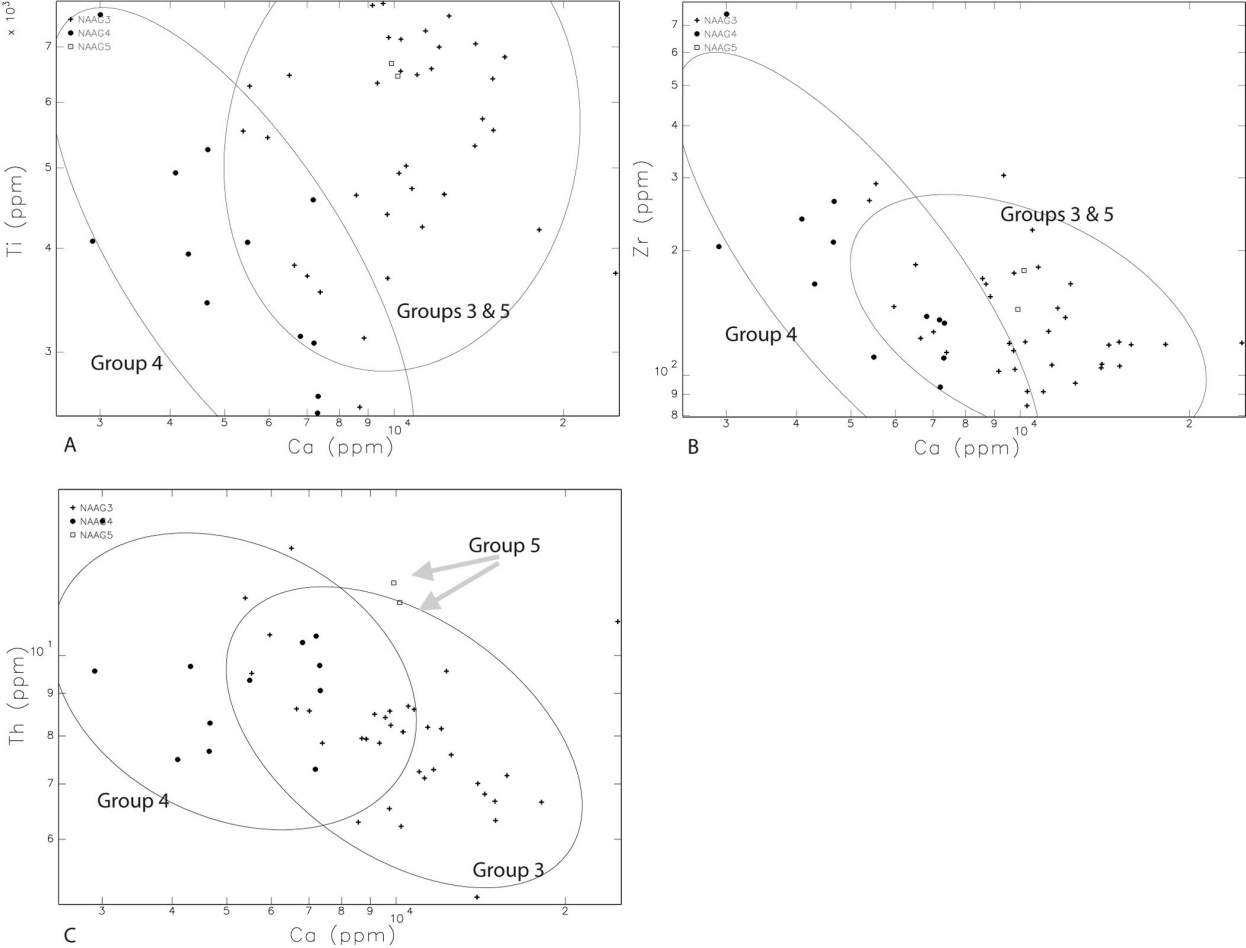
Bivariate plots A and B show that Group 4 and Groups 3 and 5 are discriminated with some overlaps. “A” has Ca and Ti and “B” has Ca and Zr. In bivariate plot C, Group 5 is discriminated from the bulk of Group 3 and Group 4.

Samples assigned to Group 3 (n = 23), Group 4 (n = 12) and Group 5 (n = 2) are listed in Table 1. Differentiating Groups 3, 4, and 5 is more difficult. However, despite some overlaps in geochemical values, Ca tends to discriminate Group 4 from Groups 3 and 5, when Ca is plotted against Zn, K, Sc, V (Fig 6A to 6D), and Ti, and Zr (Fig 7A and 7B). In the one-way analysis (S1A–S1C Fig), Group 3 has overlapping but somewhat higher values of Sc, Sr, and Ca than in Group 4. Group 3 additionally is overlapping but has somewhat lower K than Group 4 (Fig 5F). Also, in the one-way analysis (S1D Fig), except for FIP043 and 044 of Group 3, Group 5 is higher in Fe than other samples from Groups 3 and 4. Group 5 additionally has higher Ta than other samples in Groups 3 and 4, except for FIP032 in Group 3, and FIP055 in Group 4 (S1E Fig). Group 5 is discriminated from Group 4, containing inclusions associated with sandstones observed with petrography, with higher V (S1F Fig) and Ca (S1B Fig) in the one-way analysis while this is not the case with Group 3 with a wide distribution of V and Ca (S1F and S1B Fig). Group 5 has more elevated As except for FIP055 (Fig 5A), and Cr, Th, and Ti, except for FIP055, than Group 4 (S1G–S1I Fig). Finally, the bivariate plot of Ca and Th slightly discriminates Group 5 from Group 3 and Group 4 (Fig 7C).

In summary, without the elimination of any of the 58 samples, the one-way and bivariate plots are sufficient to differentiate the five identified compositional groups with assignments listed in Table 1. Although Groups 3, 4, and 5 are less precisely discriminated compared to Groups 1 and 2, Group 4 can be distinguished from Groups 3 and 5, with some geochemical overlaps. Group 5 can be discriminated from Groups 3 and 4 with selected elements. The small size of the groups prevents the robust calculation of group membership probabilities using Mahalanobis Distance calculations [86].

## Discussion

As hypothesized, our results from the bulk geochemistry corroborate with the results from the petrographic analyses. This study clearly differentiates Group 1, Group 2, and Groups 3 to 5, the equivalent of petrographic Type 1, Type 2, and Types 3 to 5 (Table 1). Petrographic Type 1 with an AT volcanic glass-rich composition is assigned as a possible non-local. Petrographic Type 2 with bedrock-derived granitic rocks and associated minerals are confident non-local. Types 3 to 5 are assigned as local sherds. By NAA, clearly discriminating Types 3 to 5 is difficult but there are some subtle geochemical differences. By petrographic analysis, Types 3 and 5 tend to have similar rock and mineral compositions but in distinct proportions. Mineral and rock fragments likely derived from sandstone in Type 4 differed more clearly from Types 3 and 5. The NAA geochemistry supports these results.

Arsenic is an unusual element to be used as the discriminator of pottery groups. In Group 1, most geochemical studies made on the volcanic glass of AT tephra with the electron microprobe were major oxides SiO_2_, TiO_2_, Al_2_O_3_, FeO, MnO, MgO, CaO, Na_2_O, and K_2_O. On the other hand, NAA in this study provides the bulk quantitative geochemistry of 32 elements. Therefore, this study adds a hypothesis that the volcanic glass and/or clayey sediment from Aira Tn Tephra (AT) has elevated As concentrations, compared to local clayey sediments from Tanegashima, and the mixture of igneous rocks and associated minerals derived from volcanic eruptions, and sedimentary rocks and associated minerals. Additionally, As is known to be a mobile element and the conditions for mobility in sediments have been tested with results including the As mobility associated with the Fe content (e.g., [87–89]). There is a possibility that the AT volcanic glass-rich paste of Group 1 adsorbs more As than samples with distinct petrographic and paste compositions from Groups 2 to 5. The adsorption may have occurred after deposition at Sankakuyama I. However, Group 1 samples were excavated from distinct grids within Area A of Sankakuyama I (Table 1, and grids shown in Fig 2) and that sample FIP001 is found in the same area of the site from where other Group 3 samples (FIP002 to 005 and 007) were excavated with FIP001 of Group 1 being the only sample with elevated As (S1 Table). A particular depositional condition should not be the main reason for the high As in Group 1 samples. For this reason, more tests, a larger sample size, and a study of raw material geochemistry and diagenesis related to As are required to suggest the usefulness of As the distinguishing element of AT tephra-rich samples.

The volcanic glass-rich Group 1 also tends to have elevated Al concentrations (mean: 130184 ppm) (Fig 5C and 5D, Table 2). We hypothesized that Al should be elevated in the bedrock-derived granite and K-feldspar rich samples of Group 2; however, Group 2 (mean, 87139 ppm), not only has lower Al than Group 1 but also has somewhat lower concentrations on average than the likely locally produced sherds of Group 3 (mean, 10386 ppm), Group 4 (mean, 88637 ppm), and Group 5 (mean, 105359 ppm) (Fig 5C and 5D, Table 2). Groups 3 and 5 have mixed sedimentary and pyroclastics materials, and Group 4 has sandstone-like inclusion compositions. The much higher Al in Group 1 and somewhat higher or similar Al rage in Groups 3 to 5 compared to Group 2 suggests that the clayey sediment matrix of Group 1 likely is elevated in Al and similarly, those of Groups 3 to 5 have similar concentrations of Al in the paste matrix or rocks and mineral inclusions combined are not too low in Al unlike the earlier assumption.

As expected, our bulk geochemistry on granitic sherds also parallels the results from the petrographic study because Group 2 pottery rich in granitic bedrock-derived inclusions has exceptionally high K compared to other sherds shown both in the one-way analysis and bivariate plots (Fig 5B, 5D and 5F). Other elements in Group 2, however, sometimes have larger coefficient of variation (Table 2). This suggests that the raw materials of these samples may be from distinct granitic units from the Osumi Peninsula and/or Yakushima [41]; nonetheless, we do not subclassify these samples into distinct geochemical groups in this study as our sample size is too small. The geological reasons for why there is the tendency of elevated Rb, Ta, and Th in Group 2 than in other groups (Fig 5E, 5G and 5H) is unknown.

We do not attempt to geochemically subdivide Group 3 with frequently widely ranged elemental values within the group. Even petrographically, although it is dominated by single grains of phenocrysts with lesser amounts of sedimentary materials [41], each sample is unique having distinct amounts and compositions within the large number of single grains of phenocrysts, sedimentary grains found in lesser amounts, and trace amounts of plutonic rock fragments that possibly come from Yakushima via sea currents, and trace amounts of plutonic phenocrysts derived from pyroclastics [41]. These non-uniform inclusion characteristics suggest that producers were not procuring clayey sediments with or without added inclusions from a single location. Also, different potters may have procured raw materials from distinct locations, or within the several hundred to over a thousand years of the Incipient Jomon, raw material gathering locations changed. We expect that future research on the Incipient Jomon pottery from other sites on Tanegashima will help characterize and identify the variability in the paste recipe of Group 3. Group 4 has smaller elemental compositional variability when compared to Group 3, but it also has some level of variability. This result corroborates with the observation from petrographic Type 4 inclusions suggested to have been derived from sandstone-related rocks and minerals but as not having a homogeneous composition. Group 4 materials, therefore, are not likely to have been gathered from a homogeneous deposit or from a single location. Group 5 is a small sample (n = 2), similar in inclusion types to Group 3 but with even amounts of single grains of phenocrysts and sedimentary rocks. It is expected to be more geochemically uniform than Group 3, however, further studies with increased sample size are necessary to geochemically characterize this tentatively defined group.

The overall outcome strongly supports the petrographic results suggesting that pottery assigned to Groups 3, 4, and 5 are locally produced ware with raw materials available in the vicinity of Sankakuyama I (e.g., [41]). Samples from each of Group 3 and 4 are not likely from uniform deposits creating petrographic and geochemical variability within. The sample size of Group 5 is too small to project the variability within the group. Group 1 sherds are likely non-local wares. As this group is produced with AT tephra-based sediments, they are probably from the northern part of the Osumi Peninsula, the AT-tephra dense area. Group 2 is clearly discriminated by K concentrations and this parallels the results from petrography showing fresh and angular granitic rocks rich in K-feldspar that are likely from the Osumi Peninsula or Yakushima. Understanding of possible spatial distribution of source types within the site would require an increased sample size. Finally, this study is another case that suggests, for the sourcing of earthenware pottery, combining petrographic and bulk geochemical analysis yields effective results.

## Conclusions

At Sankakuyama I, there are various signatures of decreased mobility such as a large ceramic assemblage of about 4000 sherds, heavy grinding stones, polished stone axes, polished arrowheads, pithouses, and high tool diversity (e.g., [56, 90–94]). Combining NAA geochemical results with the suggestions from petrographic analysis in Iizuka et al. [41], a large proportion of pottery, in our study about 86–90%, is likely locally produced ware, suggesting that hunter-gatherers occupying the site had high degrees of sedentism. In this study, the approximately 10% of confidently foreign vessels suggests they are from long-distance exchanges. Either local resources were contained in pottery and exchanged and/or pottery itself may have functioned as the commodity (modifying [41]). Some degrees of long-distance exchange are expected as risk buffering behaviors for foragers residing in areas with resource predictability and concentration [77, 78]. If the ceramics are from granitic batholiths zones of Yakushima or the southern Osumi Peninsula, and northern Osumi, it is unlikely residents of Sankakuyama I produced the vessels during logistical activities and transported the vessels back to the base camps of Sankakuyama I as such behaviors are costly for hunter-gatherers expected to maximize return during forays [41].

With the expectation that foragers engage in exchange with other groups in distinct resource and environmental zones in order to buffer risks [77, 78, 90, 95], network brokerage sites linking groups and people [96, 97] are suggested to exist between producer and consumer end points. Additionally, because the information and resource exchanges are among the activities in inter-band and supra band gatherings [77, 80], we suggest that the southern Osumi in Kyushu proper may have functioned as the mid-zone for exchange. The southern Osumi may have been where Group 2 pottery was produced, with other Incipient Jomon sites to the north where raw materials of Group 1 is abundant. The lack of known Incipient Jomon sites on Yakushima that may have functioned in an exchange network adds to this inference. Nevertheless, no thorough surveys have yet to be conducted and no sites have been identified on the southern side of the Osumi Peninsula facing the Osumi Strait. Only with future studies on clear distinctions of production zones between the southern Osumi and Yakushima batholiths and the discovery of sites should we able to provide answers.

Putting the origins of pottery in the context of the terminal LUP to Incipient Jomon transition, the degrees of exotic lithic use from the terminal LUP (estimated within the range of ca. 17,000/16,000–14,000 cal BP in the Oldest Dryas, colder condition to the beginning of the Bølling-Allerød [64]) and the exact timing of change to the Incipient Jomon are yet to be investigated. However, the microblade use, low tool variability, smaller number of sites, and a lack of substantial features on Tanegashima (e.g., [57, 61]) suggest a high residential mobility for the terminal LUP. This means, there was a significant behavioral reorganization toward increased sedentism, and adoption of a variety of new tools and features in the Incipient Jomon (e.g., [56]) with the regular incorporation of pottery use. An ecotone condition with possible concentrated and predictable resources in the warmer Bølling-Allerød continued from the LGM on Tanegashima likely corresponding with significantly increased degrees of sedentism in the Incipient Jomon. The change may correspond with increased occupations and sea level rise disconnecting lands.

Obsidian artifacts on Sankakuyama I from the Incipient Jomon clearly come from Kyushu proper. Assumptions have been made that obsidian tools include those from the Kuwanokizuru-related source(s) from the Kumamoto Prefecture of central, and Himejima Island of northeastern Kyushu proper [62]. The direct or indirect long-distance circulation extended to about 330 km. This can indicate that after the sea level rise and the disappearance of the land bridge between Tanegashima and Kyushu proper by ca. 14,300 cal BP (e.g., [55]), sedentary foragers began to engage in long-distance exchange of small flake-based tools (perhaps newly) involving a costly ocean navigation. Small exotic flake-based tools may have been exchanged with greater frequency, but at this point we do not know the mechanisms of exchange and differences in exchange intensity between pottery and small flake-based tools.

Tanegashima pottery adopted in the Bølling-Allerød was produced and used by foragers with increased sedentism in a context of abundant resources. Exchange was the means of long-distance pottery circulation. The vessel adoption is observed with drastic behavioral reorganizations from the earlier highly mobile foraging of the pre-ceramic, terminal LUP. This, however, may differ from Kyushu proper. There, probable small amounts of pottery that may have appeared by ca. 15,000 cal BP in the context of microblade production and circulation patterns is yet to be understood. This paper demonstrates the first example which systematically reconstructs the pre-Younger Dryas pottery production and exchange within East and Northeast Asia where the first ceramic vessel production occurred. The result is obtained in the context of a well-documented artifact and features and an unusually firm tephrochronology within these regions, allowing the reconstruction of pottery related behavior in the Bølling-Allerød. Although the study is just from one site, this is the first NAA-based sourcing done on the late Pleistocene pottery from the Japanese archipelago which confirms the results from petrography. In the future, we should compare our results by increasing sample sizes, number of sites, and by further refining the high-resolution geochronology for finer scale reconstruction of production and circulation, to make better inferences on degrees of mobility, sedentism, and exchange.

## Supporting information

S1 Fig Plots of one-way analysis. These plots show geochemical composition of samples in Goups 3 to 5 plotted with Sc in “A”, Ca in “B”, Fe in “C”, Sr in “D”, Ta in “E”, V in “F”, Cr in “G”, Th in “H”, and Ti in “I”. “G” in the X-axis stands for group.
(TIFF)

S1 Table Raw geochemical quantitative data obtained by NAA and adopted in this study. (XLSX)
