# Supplementary figures and images for: Late Pleistocene pottery production and exchange: Provenance studies of hunter-gatherer wares from southern Kyushu, Japan by neutron activation analysis
Source: PLoS One. 2022 Mar 16;17(3):e0265329. doi: 10.1371/journal.pone.0265329 (PMC8926207; doi:10.1371/journal.pone.0265329)

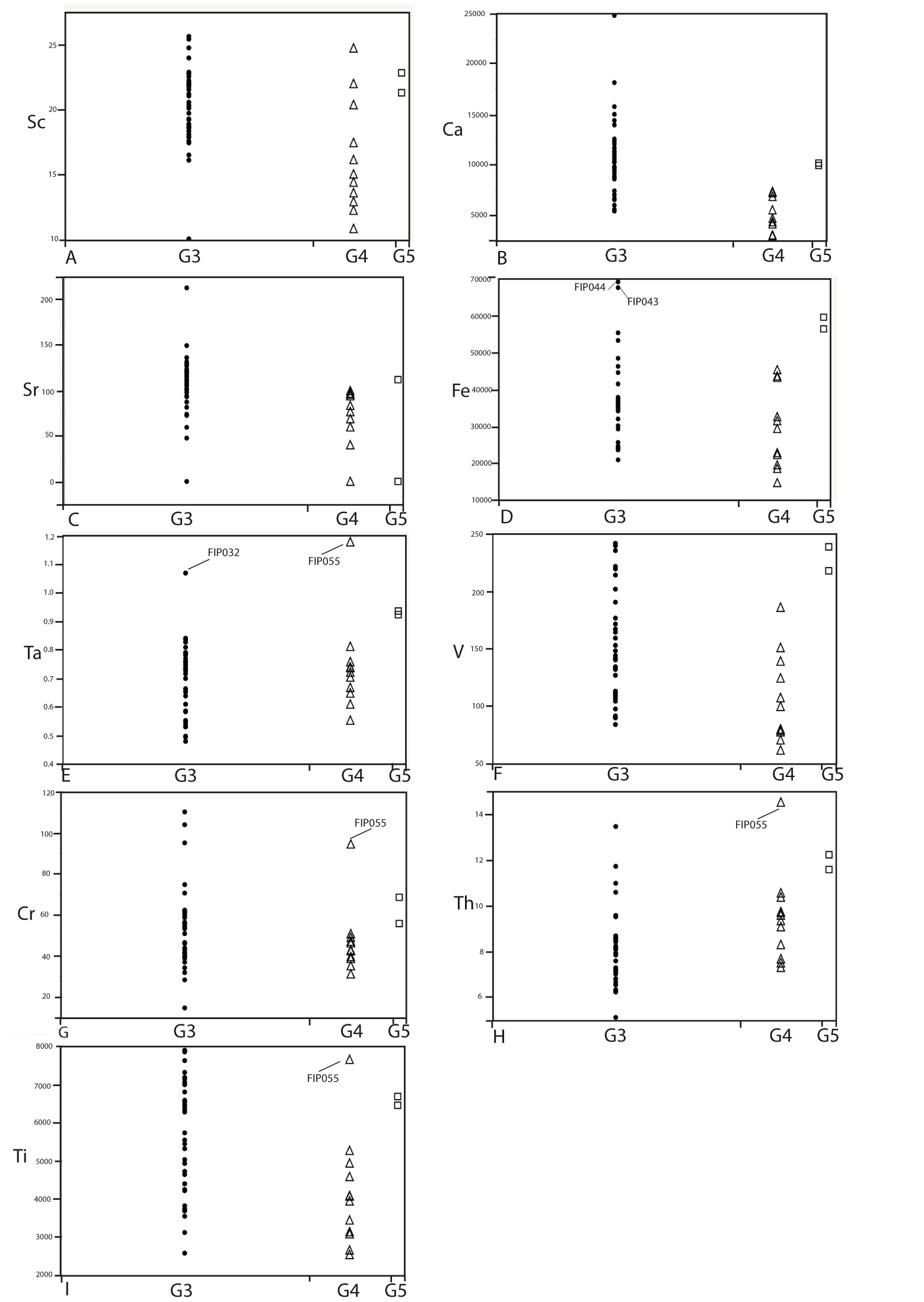

Supplement: S1 Fig — These plots show geochemical composition of samples in Goups 3 to 5 plotted with Sc in “A”, Ca in “B”, Fe in “C”, Sr in “D”, Ta in “E”, V in “F”, Cr in “G”, Th in “H”, and Ti in “I”. “G” in the X-axis stands for group. (TIFF) [file pone.0265329.s001.tiff]
